# Neural network and spline-based regression for the prediction of salivary hypofunction in patients undergoing radiation therapy

**DOI:** 10.1186/s13014-023-02274-9

**Published:** 2023-05-08

**Authors:** Derek K. Smith, Haley Clark, Allan Hovan, Jonn Wu

**Affiliations:** 1 American Dental Association Science and Research Institute, 211 E Chicago Ave., Chicago, IL 60611 USA; 2 Medical Physics; BC Cancer, Surrey, BC Canada; 3 BC Cancer, Vancouver, BC Canada; 4 Department of Radiation Oncology, BC Cancer, Vancouver, BC Canada

**Keywords:** Machine learning, Neural networks, Salivary hypofunction, Normal tissue complication probability

## Abstract

**Background:**

This study leverages a large retrospective cohort of head and neck cancer patients in order to develop machine learning models to predict radiation induced hyposalivation from dose-volume histograms of the parotid glands.

**Methods:**

The pre and post-radiotherapy salivary flow rates of 510 head and neck cancer patients were used to fit three predictive models of salivary hypofunction, (1) the Lyman-Kutcher-Burman (LKB) model, (2) a spline-based model, (3) a neural network. A fourth LKB-type model using literature reported parameter values was included for reference. Predictive performance was evaluated using a cut-off dependent AUC analysis.

**Results:**

The neural network model dominated the LKB models demonstrating better predictive performance at every cutoff with AUCs ranging from 0.75 to 0.83 depending on the cutoff selected. The spline-based model nearly dominated the LKB models with the fitted LKB model only performing better at the 0.55 cutoff. The AUCs for the spline model ranged from 0.75 to 0.84 depending on the cutoff chosen. The LKB models had the lowest predictive ability with AUCs ranging from 0.70 to 0.80 (fitted) and 0.67 to 0.77 (literature reported).

**Conclusion:**

Our neural network model showed improved performance over the LKB and alternative machine learning approaches and provided clinically useful predictions of salivary hypofunction without relying on summary measures.

## Background

Radiotherapy to the head and neck can result in a wide array of morbidities. One such morbidity, which can have a devastating effect on oral health, is radiation-induced salivary gland hypofunction [1]. Despite being a relatively slow-dividing type of cell, salivary glands demonstrate remarkable sensitivity to ionizing radiation. At low doses radiation damage may be reversible, but substantial decreases in salivary function have been noted with even moderate doses of radiation (30–40 Gy) [2, 3]. Cumulative therapeutic doses of radiation of 60–70 Gy often result in irreversible loss of function.

Saliva has diverse function in the oral cavity. Saliva moistens the oral mucosa, contains antibodies that facilitate immune response to oral microbes, buffers against changes in pH, contains minerals that allow for remineralization of damaged tooth structure, cleanses the teeth, moistens food to aid in swallowing, and is required for normal gustatory sensation. HNC survivors that experience hyposalivation consequently are at risk for an increase in dental disease, fungal infection, dysphagia, dysgeusia, and friability of the oral mucosa. Because these symptoms interfere with eating, taste, and speech they can contribute to social isolation and poor quality of life [4, 5].

Predicting salivary gland hypofunction after radiation therapy is complicated by the sophistication of modern radiation plans. The most common way to summarize these plans is using a dose-volume histogram (DVH), which is a step function relating the dosage (ranging from 0 to the maximum dose) and the fraction of the total organ volume that received at least the given dose. The DVH requires processing to be incorporated in predictive models as it has a high number of dimensions. Since its development in the 1980s, the Lyman-Kutcher-Burman (LKB) model has been the standard method for processing the DVH and assessing normal tissue complication probabilities (NTCP) during radiation treatment planning. This model is appealing in that it is estimated using parameters that are tangible, volumes of the organ in question, the dose that would result in 50% NTCP with uniform irradiation of the total volume (${TD}_{50}(1)$), an organ specific parameter describing how the organ is affected by partial irradiation (n), and the slope of the dose–response curve (m). Despite the clinical appeal of this class of models, they have a particular limitation as they often are not well calibrated, a criticism that was recognized by the authors in their original publication [6]. Another limitation is that the LKB model requires a binary input for what constitutes a complication necessitating an arbitrary cutoff to be established for the fitting process. Finally, the LKB does not allow for inclusion of other potentially relevant covariates. While this may be a lesser concern in radiation planning specifically, having a model that incorporates the radiation treatment information separate from any model that incorporates information on comorbidities and medications presents a problem when trying to holistically assess a patient’s risk of developing a complication. The LKB model has been applied to the parotid glands and data exists which estimate relevant parameters for model fitting [7].

The LKB model was the original prediction model for NTCPs and was applied to a wide variety of organs. Roesink et al. [7] performed a study in 2001 in which they used a cohort of 108 patients to estimate the parameters of the LKB for parotid glands and salivary hypofunction. More recently, Beetz et al. [8] and Li et al. [9] developed regression models that predict salivary hypofunction using a variety of clinical and demographic information in addition to a DVH-derived mean organ dose in cohorts of size 167 and 365 respectively. Still others have attempted to predict either xerostomia or salivary hypofunction without using any data on the radiation plan [10, 11] while others have turned to delta radiomics [12] for prediction, although these calculations cannot be performed in the radiation planning phase.

Among these attempts, the only studies that utilized the radiation plan as a predictor (the LKB model via Roesink and the regression-based models of Beetz and Li which relied on mean organ dose), only utilized a gross summary of the DVH. In this manuscript, we compare the LKB method for assessing the probability of radiation induced salivary gland hypofunction to two alternative methods of prediction, which incorporate the information from the radiation DVH in a way in which the full information about the distribution of radiation dose across the organ is preserved. The first method summarizes the DVH using a cubic spline basis and uses this as input to a standard regression model. The second method registers the value of the DVH at each dose and uses these as inputs into a neural network. This study arrived at new estimates of the LKB model parameters for the parotid glands which were derived in a larger cohort than those previously reported, developed and demonstrated two methods for incorporating the complete information contained within the DVH into a prediction model, and obtained some level of evidence that grossly summarizing the radiation plan may adversely impact prediction of NTCPs in the parotid glands.

## Methods

### Patients

510 patients undergoing radiotherapy for H&N cancers at BC Cancer between November 2004 and July 2015 were enrolled in this study. Patients were treated with either intensity modulated radiation therapy or volumetric modulated arc therapy. Radiation dose for all radiotherapy plans were calculated using the analytical anisotropic algorithm using the same planning system, dose prescribing convention, and dosimetric grid size. DVHs for each patient’s parotid glands (both ipsilateral and contralateral to the tumor site) were extracted using DICOMautomation. Patients were excluded if: they were unable to follow written saliva collection procedures; they received atypical chemotherapy agents (i.e. an agent other than cetuximab, cisplatin, carboplatin, or gemcitabine); they received electron therapy; or they had previous interfering radiotherapy. In addition to routine clinical quality assurance procedures prior to delivery of radiotherapy plans, a single senior H&N Radiation Oncologist (JW) validated the consistency and accuracy of salivary contours of the parotid glands specifically for research quality assurance purposes after plan delivery. Although salivary function can also be impacted by radiation to the other major salivary glands, stimulated salivary function is most impacted by the parotid glands. In addition, the LKB model requires gland specific parameters and therefore cannot be fit or otherwise compared to the candidate approaches in a mixed-gland framework.

### Saliva collection

Stimulated saliva was measured prior to radiotherapy and one year following completion of treatment. Measurements comprise whole-mouth saliva collected with patients in prone or upright position over a five-minute period while chewing flavorless wax.

### Radiation planning/data collection

All clinical plans were created according to institutional guidelines using the Varian Eclipse treatment planning system. Dose-volume histograms for the parotid glands were extracted from clinical plans using DICOMautomaton [13], an open source toolkit for radiotherapy analysis.

### LKB model

The LKB model addresses the multidimensionality of a DVH as a predictor variable by reducing it to a single dose and volume in a process originally described by Lyman [6, 14]. This reduction of the multidimensional DVH to a single dose over an effective volume is justified by an assumed power law relationship, where $i$ represents each step of the DVH.

$$V=\sum_{i}\Delta {V}_{i}{\left[\frac{{D}_{i}}{{D}_{max}}\right]}^\frac{1}{n}$$

Transformed single-step histograms are assumed to have the same complication probability as the original one. The newly transformed dose and volume are then used to normalize the dose with $m\times {TD}_{50}(v)$ serving as an estimate of the standard deviation of dose, where V and D are the transformed values from the DVH and *V*_total_ is the total organ volume (in this case the parotid glands).

$$t=\frac{D-{TD}_{50}(v)}{m \times {TD}_{50}(v)}$$

where ${TD}_{50}(v)$ is attained from another assumed power law.

$$v=\frac{V}{{V}_{total}}$$

$${TD}_{50}\left(1\right)={TD}_{50}(v)\times {v}^{n}$$

The final model estimate is then calculated by plugging the estimated *t* into the cumulative distribution of a standard normal random variable.

$$NTCP=\frac{1}{\sqrt{2\pi }}{\int }_{-\infty }^{t}{e}^{\frac{{-t}^{2}}{2}}dt$$

Due to the LKB model requiring a binary definition of complication, patients were defined to have a complication if their post-radiation salivary flow rate was reduced to less than 25% of the preoperative rate (i.e., a ‘severe’ reduction). The model was fit using maximum likelihood. After transforming the DVHs to a single dose and volume, the ratio of post-treatment to pre-treatment whole salivary flow was dichotomized and the model fit resulting in the 3 organ specific parameters (TD_50_(1), m, n). Roesink et.al conducted a study of 93 patients in which these parameters were estimated to be 31 Gy, 0.54, and 1 respectively [7]. The fitted values as well as those reported by Roesink et al. were also used in the assessment of the candidate models fit with new methods.

### Alternative models

**Fig. 1:**
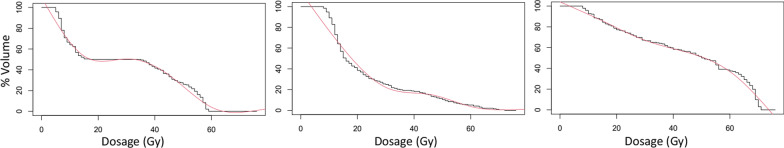
Three examples of spline approximation of dose-volume histograms and their approximation by cubic spline basis (red)

The first alternative model addresses the high dimensionality of the DVH using a cubic spline basis. This procedure fits a polynomial function with a specified form to each of the DVHs in the dataset. For this application, it was decided that a spline function with 5 knots equally spaced across the range of observed doses imparted sufficient flexibility to adequately mimic the DVHs, Fig. 1. The resulting fitted model contains six coefficients which are then used as predictors in a logistic regression model, which also incorporates splines to improve model flexibility.

The second model extracts the volume recorded in the DVH at intervals of 1 Gy from 0 to 70 Gy. These values are then used directly as inputs into a neural network with a single hidden layer containing 12 nodes and a decay of 0.8, which is a form of regularization for the model. These model parameters were determined by using ten-fold cross validation to obtain optimal predictive performance.

Both of these candidate models contain tuning parameters, which were optimized using tenfold cross-validation of the AUC for predicting a decrease in salivary flow rate of 0.5*baseline. In the case of the regression-based approach, the cubic splines were applied to the model inputs with the number of knots being tuned by tenfold cross-validation. In the case of the neural network, the decay parameter was employed. The decay parameter regularizes the model penalizing the size of the weights to prevent overfitting and improves the performance of the model-fitting algorithm by reducing flat spots in the cost function by inducing a differential penalty between highly correlated inputs like those coming from the dose-volume histogram. Additionally, alternative architectures were tested in which the number of inputs were reduced to as few as 10-equally spaced readings from the DVH and hidden layer sizes ranging from 5 to 25. However, reducing the number of inputs did not improve performance with performance being negatively effected at the smallest number of inputs.

### Evaluation

The data was partitioned into a training set containing 70% of the observations and a test set containing 30%. The predictive performances of the models were compared using area under the receiver operating characteristic curve in the test set. Sensitivity to the cutoff for reduction in salivary flow rate was examined by including a variety of other potential cutoffs. All analyses were conducted in the R statistical computing program [15].

## Results

### Patient demographics

Since salivary measurements were considered standard-of-care for dental monitoring at the study site, study participant demographics are representative of institutional-level head-and-neck radiotherapy patient demographics. 335 (65.7%) were male, 118 (23.1%) were female, and 57 (11.1%) were unknown or other gender. Average patient age when radiotherapy began was 59.8 years (standard deviation: 11.9 years; minimum: 18.8 years; maximum: 90.9 years).

Tumour primary site was: nasopharynx for 110 (21.6%) patients; tonsil for 94 (18.4%); base of tongue for 76 (14.9%); larynx for 27 (5.2%); thyroid for 13 (2.5%); and unknown or various other sites for the remaining patients (Table 1).

**Table 1:** Demographics for study participants

	n(%), mean(sd)
Sex	
Male	335 (65.7%)
Female	118 (23.1%)
Other/Unknown	57 (11.1%)
Age (y)	59.8 (11.8)
Tumor Site	
Nasopharynx	110 (21.6%)
Tonsil	94 (18.4%)
Base of Tongue	76 (14.9%)
Larynx	27 (5.2%)
Thyroid	13 (2.5%)
Other/Unknown	190 (37.2%)

### Salivary function

Patients baseline salivary function was measured prior to initiation of cancer therapy. The mean whole stimulated salivary flow rate was 1.47 g/min (95% CI (1.40, 1.55)). Post cancer therapy, salivary function was reassessed at 3 months and one year post radiation, with the one-year data being used to train the model. The post therapy mean whole stimulated salivary flow rates were 0.57 g/min (95% CI (0.52, 0.62)) and 0.77 g/min (95% CI (0.70, 0.83)) respectively.

### Model fitting

The resulting LKB model parameter estimates from the data were 39.2 Gy, 1.1, and 1 for TD_50_(1), m, and n respectively. During the fitting of the candidate machine learning models, the number of inputs for the learning methods was considered. Models were fit using the ipsilateral parotid data only as well as combining data from the ipsilateral and contralateral glands. Given no substantial improvement in predictive ability, the data presented here represents the more parsimonious approach, which utilized only data from the ipsilateral parotid gland.

### Comparison of predictive ability

**Fig. 2:**
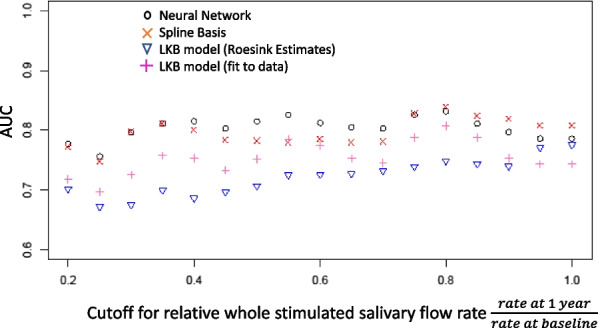
Cutoff dependent AUC for 4-candidate models: This figure illustrates how the four models compared to each other depending on what cutoff was chosen to define a salivary function complication. For example 0.5 on the x-axis indicates the results obtained when patients whose salivary function decreased by half or more were considered a complication

The LKB, neural network, and the spline basis models were fit in the training set and predictions were derived for the test set. Predictions based on the parameter estimates found by Roesink et al. were also generated for the test set. Because model performance may differ depending on the definition of what constitutes a complication, predictive accuracy was evaluated using a cutoff dependent area under the ROC curve. The neural network model dominated the LKB models demonstrating better predictive performance at every cutoff with AUCs ranging from 0.75 to 0.83 depending on the cutoff selected. Similarly the spline based model nearly dominated the LKB models with the fitted LKB model only performing better at the 0.55 cutoff. The AUCs for the spline model ranged from 0.75 to 0.84 depending on the cutoff chosen. The LKB models had the lowest predictive ability with AUCs ranging from 0.70 to 0.80 (fitted) and 0.67 to 0.77 (Roesink et al.), Fig. 2.

The statistical significance of the difference between the models was dependent on the cutoff chosen to define the complication. Using a cutoff at 0.5*baseline, all differences were statistically significant ANN vs regression (*p* = 0.004); ANN versus LKB (*p* < 0.001); regression versus LKB (*p* = 0.031). The neural network maintained statistically significant p-values at cutoffs from 0.45*baseline to 0.7*baseline. Below 0.45*baseline and at 0.7–0.75*baseline, there was no significant difference between the regression and neural network’s performance. The difference between the neural network and the fitted LKB model was statistically significant at every cutoff. The regression approach was statistically superior to the LKB model at all cutoffs with the exception of 0.55–0.60*baseline at which point they performed similarly.

## Discussion

Since the original formulation of the LKB model, its limitations have been recognized [6]. However, the convenient and clinically relevant parameterization of the LKB model coupled with a lack of compelling alternatives have made it a mainstay of radiation treatment planning. Although these parameters have proved to be clinically useful, they are only directly comparable between organs to the extent that the LKB model assumptions are uniformly satisfied for each. Parameters in the alternative approaches lack a readily apparent clinically relevant interpretation. In order to obtain similarly relevant organ specific information, the user must analyze how the predictions from the model change with varying inputs. For example, feeding a DVH representative of uniform irradiation at various levels can be used to determine TD_50_ by simply inputting dosage until the model returns a 50% complication probability. The effect of partial irradiation, typically associated with LKB parameter *n*, could similarly be determined by the feeding the models DVHs consistent with partial irradiation. Unlike the LKB model estimates, clinically useful measures obtained in this manner would likely be directly comparable under any circumstances.

Comparison of the predictive ability of the four candidate models suggests that alternative approaches to incorporating dose-volume histograms paired with modern machine learning approaches can provide improved discrimination for the prediction of post-radiation hyposalivation. These improvements are likely due to information loss in the way in which the LKB model and similar approaches which rely on simple low-dimensionality summaries of the DVH incorporate the DVH information, such as average organ dose [8, 9].

Minor differences between the LKB model parameters in the fitted LKB model and the model from Roesink et al. can likely be attributed to a combination of estimation error and differences in measuring and defining salivary hyposalivation. These differences also account for the model’s low performance. The Roesink study was relatively small (n = 108) resulting in a relatively large degree of random error. In addition, effective LKB parameter values may shift over time due to advances in therapeutic methodologies.

Models which start with the DVH are limited by the fact that they cannot account for the variable clinical impact of radiation delivered to specific anatomic regions; spatial location information is lost when the radiation is summarized as a DVH. There is some evidence to suggest that radiation to specific regions of the parotid glands, for example those containing stem and progenitor cells, may be particularly detrimental [16–18]. This limitation would apply to any DVH-based approach to an organ with such a region, where functional capacity was particularly dependent on a specific anatomical location. However, in these cases similar machine learning models could be applied directly to the three-dimensional dosimetry data, but these approaches would require extra steps to achieve spatial registration and extremely large amounts of data, likely on the order of tens of thousands of patients, to properly fit the predictive models.

## Conclusions

One advantage of the alternative modeling approaches explored here is that they can easily incorporate other clinical information relevant to predicting patient outcomes. Although this study was limited by the lack of availability of other clinically meaningful information, future studies could incorporate other factors pertinent to predicting hyposalivation such as chemotherapies, comorbidities, use of other medications commonly associated with hyposalivation, and delta radiomics data, which have been shown to have predictive value beyond that of dosing information alone [19–21]. In addition, this study was limited by lack of access to the DVHs of other major salivary glands. However, it is unlikely that the addition of the information from these glands would have made a substantial difference in predictive ability as they almost certainly contain less predictive information than is contained in the contralateral parotid gland, whose addition did not improve predictive accuracy. It is possible that information about the other glands could prove more fruitful in even larger cohorts. While the model using the DVH alone is helpful for radiation planning, larger models that can accurately predict complications from any cause could help supportive care teams to quickly and accurately identify side effects and intervene proactively.

## Data Availability

Data access can be requested in collaboration with the BC cancer institute.
